# All pure bipartite entangled states can be self-tested

**DOI:** 10.1038/ncomms15485

**Published:** 2017-05-26

**Authors:** Andrea Coladangelo, Koon Tong Goh, Valerio Scarani

**Affiliations:** 1 Department of Computing and Mathematical Sciences, California Institute of Technology, 1200 E California Boulevard, Pasadena, California 91125, USA; 2 Centre for Quantum Technologies, National University of Singapore, 3 Science Drive 2, Singapore 117543, Singapore; 3 Department of Physics, National University of Singapore, 2 Science Drive 3, Singapore 117542, Singapore

## Abstract

Quantum technologies promise advantages over their classical counterparts in the fields of computation, security and sensing. It is thus desirable that classical users are able to obtain guarantees on quantum devices, even without any knowledge of their inner workings. That such classical certification is possible at all is remarkable: it is a consequence of the violation of Bell inequalities by entangled quantum systems. Device-independent self-testing refers to the most complete such certification: it enables a classical user to uniquely identify the quantum state shared by uncharacterized devices by simply inspecting the correlations of measurement outcomes. Self-testing was first demonstrated for the singlet state and a few other examples of self-testable states were reported in recent years. Here, we address the long-standing open question of whether every pure bipartite entangled state is self-testable. We answer it affirmatively by providing explicit self-testing correlations for all such states.

## Abstract (web summary)

Device-independent self-testing is an approach that allows a complete certification of an unknown quantum state, simply by inspecting outcomes of measurements. Here, the authors demonstrate that any pure bipartite entangled state can be self-tested.

Since it was proposed a decade ago^1^, the device-independent certification of quantum devices has attracted a lot of interest because it requires minimal assumptions: the no-signalling constraint on the devices, and the validity of quantum theory. If these are accepted, the certification can then be performed by a purely classical user, that queries the devices with classical inputs and observes the correlations in the classical outputs. This is possible thanks to the violation of Bell inequalities^2^, and constitutes the operational interpretation of this phenomenon.

In a device-independent way, one can bound specific quantities like the amount of randomness^3^, the length of the secret key in quantum cryptography^1^ or the dimension of the Hilbert space of the systems involved^4^. But for some correlations, the characterization can be as complete as one can hope for. Indeed, certain correlations can be achieved exclusively by measurements on a unique quantum state (up to local transformations). We adopt the technical term ‘device-independent self-testing' to refer to such a certification. Self-testing correlations can be thought of as a classical fingerprint of a state.

The fact that a purely classical user can certify the quantum state of a system is in contrast with the usual quantum state tomography, which relies on the characterization of the degrees of freedom under study and the corresponding measurements. In this case, a classical user lacking knowledge of the inner workings of a quantum device would have no choice but to trust that it has been manufactured according to specifications.

The most celebrated example of a state that can be self-tested is the maximally entangled pair of qubits (the ‘singlet' state). One self-testing criterion for this state is the maximal violation of the well-known Clauser-Horne-Shimony-Holt (CHSH) inequality^5^,^6^. Another criterion was put forward by Mayers and Yao, in the paper which coined the term ‘self-testing'^7^. Since then, self-testing of the two-qubit singlet has been made robust^8^, extended to sequential^9^ and parallel certification of many copies^10^,^11^,^12^,^13^,^14^,^15^, and its complete set of self-testing criteria with two dichotomic measurements has been provided^16^. A variety of other quantum states have also been proved to be self-testable: all partially entangled pure two-qubit states^17^,^18^, the maximally entangled pair of qutrits^19^, the partially entangled pair of qutrits that violates maximally the CGLMP_3_ inequality^20^,^21^ and a small class of higher-dimensional partially entangled pairs of qudits, through results in parallel self-testing^12^. For the multi-partite case, self-testing is known for the family of graph states^22^,^23^ and for a few non-graph three-qubit states^23^,^24^. Hence, it is clear that self-testing is not an exclusive characteristic of maximally entangled states nor qubit states. However, little is known about self-testing of higher-dimensional entangled states (that is, states of entangled qudits for *d*>2).

In this work, we prove that all pure bipartite entangled quantum states can be self-tested, by constructing explicit correlations built on the framework outlined by Yang and Navascués^17^.

## Results

### The 3*d*4*d* Bell scenario

**Figure 1:**
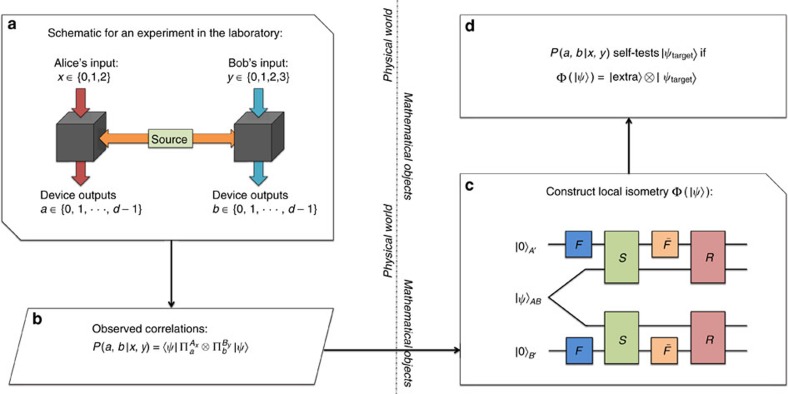
The scheme of self-testing. (**a**) First, measurement inputs and outputs from a Bell experiment in a laboratory are recorded. (**b**) Using the recorded experimental data, one can estimate the correlations of the Bell experiment. (**c**) A local isometry Φ is constructed mathematically, as in the circuit diagram. Gates *F* and 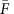 in this diagram denote the quantum Fourier transform and inverse quantum Fourier transform respectively. Gates *R* and *S*, which act jointly on 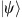 and the ancillary system, are controlled unitaries defined precisely in the Supplementary Methods. (**d**) If one can show, using the correlations, that the local isometry is such that 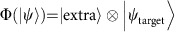, then we conclude that the correlations self-test 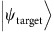.

We work in a bipartite Bell scenario, and we refer to Alice and Bob as operating the uncharacterized devices (or rather as the devices themselves). They receive inputs *x* and *y*, respectively, from the classical verifier, corresponding to their choice of measurement settings, and they return outcomes *a* and *b* respectively. In the particular scenario that we will consider, Alice has three possible measurement settings and Bob has four, while they have *d* possible outcomes each. So the inputs are *x*∈{0, 1, 2} and *y*∈{0, 1, 2, 3} and the outputs are *a*, *b*∈{0, 1, 2, ⋯, *d*−1}. We refer to this as a [{3, *d*},{4, *d*}] Bell scenario (Fig. 1a). The result of this Bell experiment can be fully described by the probabilities *P*(*a*, *b*|*x*, *y*) of obtaining a pair of outcomes *a*, *b* on measurement settings *x*, *y*. In the device-independent approach, the dimensionality of the measured system is not bounded *a priori*. Hence, the measurements made on the system can be assumed to be projective, with 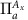 the projection corresponding to Alice obtaining outcome *a* on measurement setting *x*, and likewise for 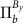 on Bob's side. No further characterization of either the state or the measurements is required, and estimating the *P*(*a*, *b*|*x*, *y*) is all that has to be done in the lab.

### Self-testing of all pure bipartite entangled states

We state our main theorem.

Theorem 1: for every bipartite entangled state of qudits 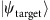, there exist [{3, *d*},{4, *d*}] quantum correlations that, when reproduced by Alice and Bob through local measurements on a joint state *ρ*, imply the existence of a local isometry Φ such that 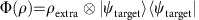, where 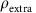 is some auxiliary state. Moreover, under the isometry Φ, the local measurements on *ρ* are equivalent to measurements that act trivially on 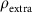 and as the ideal measurements on 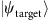 (described exactly in the Supplementary Methods).

The proof of Theorem 1 now proceeds at the mathematical level (Fig. 1c), and we provide an overview of the main ideas. The full details are contained in the Supplementary Methods. For ease of exposition, we take Alice and Bob's shared state to be a pure state 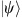, but our proof goes through in the same way for a general *ρ*. Initially, the verifier has no knowledge about the state shared by the two devices, and he wishes to certify that it is a specific state 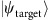 of two qudits. We can think of providing Alice and Bob with a qudit each (*A*′ and *B*′), initialized in an arbitrary state 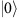; then trying to swap information from the two black-boxes into these qudits. If at the end of the swap one finds 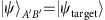, one concludes that the boxes contained the state 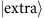
⊗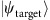 before the swap, where the precise state 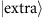 is not important, and is just ancillary. The physical and mathematical parts of self-testing are connected by the existence of a swap operation, which acts as desired thanks to the constraints given by the *P*(*a*, *b*|*x*, *y*). In mathematical terms, what we have just explained amounts to constructing a local isometry Φ such that 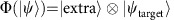. If such an isometry exists, one says that these correlations self-test 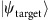. Invoking the Schmidt decomposition, self-testing all bipartite entangled states reduces to self-testing all states of the form.


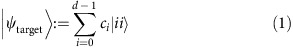


where 0<*c*_*i*_<1 for all *i* and 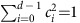.

One may wonder whether mixed states could also be self-tested, that is, if some *P*(*a*, *b*|*x*, *y*) is uniquely compatible with a mixed state (or with its purified version, but with measurements acting trivially on the purifying system). The answer is negative: any *P*(*a*, *b*|*x*, *y*) produced by a bipartite mixed state can be reproduced by a bipartite pure state of the same dimension^25^. Hence, in the bipartite scenario, the best one can hope for is to self-test every pure state. To illustrate how we construct self-testing correlations for such a target state as in equation (1), we look at the case *d*=4, so that 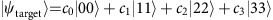. We already know that with correlations having two inputs per party, one can self-test any two-qubit state (that is, *d*=2)^17^,^18^. So, the idea is that for *x*, *y*∈{0, 1}, we choose *P*(*a*, *b*|*x*, *y*) so that the probabilities for *a*, *b*∈{0, 1} certify 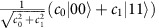, while those for *a*, *b*∈{2, 3} certify 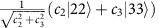. All the other *P*(*a*, *b*|*x*, *y*), that is, those where (*a*, *b*)
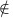
{0, 1}^2^∪{2, 3}^2^, are set to zero. Then, one similarly uses measurement settings *x*∈{0, 2} and *y*∈{2, 3}, but with a block structure certifying 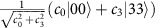 and 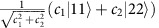.

**Figure 2:**
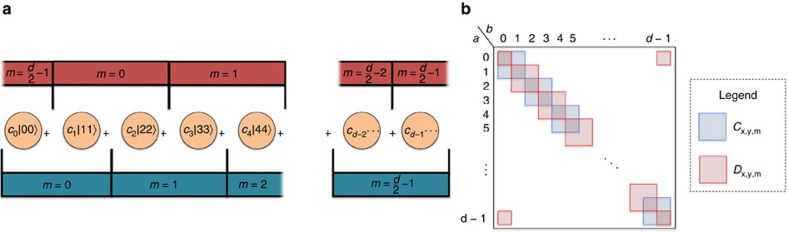
Block-diagonal correlations as two-qubit fingerprints. (**a**) In blue, the block-diagonal correlations for measurement settings *x*, *y*∈{0, 1} ‘certify' the ‘even-odd' pairs, while, in red, the block-diagonal correlations for measurement settings *x*∈{0, 2}, *y*∈{2, 3} certify the odd–even pairs. (**b**) The correlation table describes the structure of the block-diagonal correlations required for self-testing. The blocks in blue correspond to the correlations for measurement settings *x*, *y*∈{0, 1}, and the red blocks correspond to measurement settings *x*∈{0, 2}, *y*∈{2, 3}. Please refer to Supplementary Tables 1, 2, 6 and 7, for the full correlation tables.

In other words, our correlations rely on detecting a pattern of two-qubit correlations compatible exclusively with 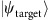, across a suitable direct-sum decomposition of the Hilbert space in which the joint state lies. The recipe is clearly not restricted to *d*=4: with the same number of measurement settings, and naturally generalized block-diagonal correlations, one can self-test any bipartite entangled pure state of any dimension (see Fig. 2 for an illustration for *d* even; the argument carries on to *d* odd as well).

### Proof outline of Theorem 1

While the recipe is intuitive, the formal proof must follow the scheme illustrated in Fig. 1, and thus construct the local isometry. All the technical details are given in the Supplementary Methods, and here we outline how the proof proceeds.

First, we need to formalize the intuition that the two-qubit blocks are certified by the block-diagonal correlations described earlier. Consider the ‘tilted CHSH' Bell-type inequality^26^


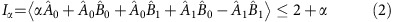


where *x*, *y*, *a*, *b*∈{0, 1}, *α*∈[0, 2), 
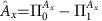
 and 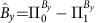. It is known, thanks to Yang and Navascués^17^, and Bamps and Pironio^18^, that maximal violation of this inequality, corresponding to 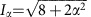, self-tests the state 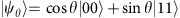, with 
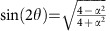
. However, when we try to apply this certification to each consecutive pair of two outcomes, we find that the value of the left hand side (LHS) of inequality (2) in each block, computed from the *P*(*a*, *b*|*x*, *y*) we described earlier, is the maximal violation multiplied by the probabilistic weight of that block: in other words, it is not the maximal violation itself. To recognize the covert maximal violation that indeed resides in each block, and the certification that follows from it, one has to realize that the state which achieves the maximal violation is not the joint state 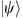, but rather its projection onto each 2 × 2 block. From each such maximal violation, one can construct the four operators 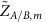, 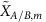, with support on the (2*m*, 2*m*+1) block (or 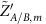, 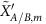 with support on the (2*m*+1, 2*m*+2) block), that are used in the self-testing isometry from Yang and Navascués^17^, and Bamps and Pironio^18^.

Second, one has to tie together the certifications in the different blocks, and explicitly construct the overall local isometry Φ such that 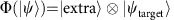. A sufficient condition for the existence of such an isometry has been formulated by Yang and Navascués^17^: one needs complete sets of orthogonal projections 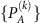 and 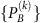 and unitary operators 
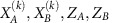
 satisfying the following conditions for all *k*=0, 1, ..., *d*−1:


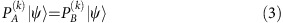



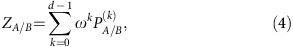



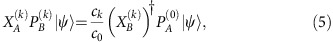


where *ω*=*e*^2*πi*/*d*^. In our construction, the projections 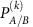 are chosen from Alice and Bob's projection measurements, and each 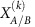 operator is the product of all the 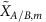 and 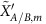 (formally extended to the whole space, and denoted 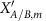 and 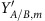 respectively in the Supplementary Methods) covering all 2 × 2 blocks up to *k*. This product spans the alternating block structure: it is in these operators that the crucial connection between blocks is encoded. It is not difficult, finally, to extend the proof of self-testing to the ideal measurements (see the Supplementary Methods).

## Discussion

In conclusion, we have proved the long-standing conjecture that all bipartite entangled quantum states can be self-tested, by explicitly providing a ‘classical fingerprint', or self-testing correlations, for every such state. Such fingerprints are not unique: our proof also remains valid if, in each block, the criterion based on the tilted CHSH inequality is replaced by any other criterion that self-tests the same two-qubit state. In particular, through the correlations adopted in Yang and Navascués^17^, a maximally entangled pair of qudits can be self-tested with only three measurements per side, that is, in the[{3, *d*},{3, *d*}] Bell scenario. We have only presented the proof of ideal self-testing (when the correlations are exact): while we believe that some robustness bounds can be derived, existing analytical tools produce notoriously unsatisfying bounds, and the numerical tools that give much better bounds can only be applied to selected examples. In this situation, we would rather wait for progress in analytical tools, of the kind shown by Kaniewski^27^.

Besides shedding new light on quantum states and quantum correlations, our result has potential applications to quantum technologies. Proofs of certification of quantum devices, from randomness to cryptography and ultimately quantum computing, have often been based on a self-testing criterion, the rigidity of the CHSH game^9^,^28^,^29^. Our work adds total flexibility of choosing the state in the bipartite scenario. One direct application may be in the context of quantum random number generation. Concretely, in device-independent randomness expansion (the first device-independent random-number generation scheme to be proposed, and the only to have been experimentally implemented to date^3^), guaranteed private randomness is generated from an initial random seed. Based on our self-testing procedure, a small random seed (two random trits) could provide up to *O*(log *d*) bits of private randomness per run, with *d* limited only by the experimental state-of-the-art. Indeed, in the ideal case, if one knows that the global state is maximally entangled, each outcome of any ideal local measurement has probability 1/*d*. A robustness analysis for self-testing both the state and the measurements is required to assess the expansion rate of any protocol based on our self-testing procedure, and we leave this for future work. Any such protocol would become feasible as soon as one can realize loophole-free Bell tests with entangled states of dimension *d*.

## 

### Data availability

Data sharing not applicable to this article as no datasets were generated or analysed during the current study.

## Additional information

**How to cite this article:** Coladangelo, A. *et al*. All pure bipartite entangled states can be self-tested. *Nat. Commun.* **8,** 15485 doi: 10.1038/ncomms15485 (2017).

**Publisher's note**: Springer Nature remains neutral with regard to jurisdictional claims in published maps and institutional affiliations.

## Supplementary Material

Supplementary Information Supplementary tables, supplementary methods and supplementary references.
