# Supplementary material for: All pure bipartite entangled states can be self-tested
Source: Nat Commun. 2017 May 26;8:15485. doi: 10.1038/ncomms15485 (PMC5458560; doi:10.1038/ncomms15485)
Supplement: Supplementary Information — Supplementary tables, supplementary methods and supplementary references. [file ncomms15485-s1.pdf]

Supplementary Information

I. SUPPLEMENTARY METHODS

In this section, we provide detailed proof of our main result.

A. The correlations and "self-testing" each block

As we mentioned in the main text, the result of a Bell experiment can be fully described by the values of the conditional probabilities, $P(a,b|x,y)$. We can arrange these $P(a,b|x,y)$ in twelve $d \times d$ correlation tables, one for each pair of measurement settings, denoted by $T_{x,y}$:

$T_{x,y} :=$

$a\backslash b$	0	1	$\cdots$	$d-1$
0	$P(0,0|x,y)$	$P(0,1|x,y)$	$\cdots$	$P(0,d-1|x,y)$
1	$P(1,0|x,y)$	$P(1,1|x,y)$	$\cdots$	$P(1,d-1|x,y)$
$\vdots$	$\vdots$	$\vdots$	$\ddots$	$\vdots$
$d-1$	$P(d-1,0|x,y)$	$P(d-1,1|x,y)$	$\cdots$	$P(d-1,d-1|x,y)$

In order to self-test the target state $|\psi_{\text{target}}\rangle = \sum_{i=0}^{d-1} c_i|ii\rangle$, where $0 < c_i < 1$, we won't need to specify the whole set of twelve correlations tables $T_{x,y}$, but it will be sufficient for us to specify the tables corresponding to measurement settings $x, y \in \{0,1\}$, and those for the settings $x \in \{0,2\}, y \in \{2,3\}$. The constraints we place on these will be sufficient to self-test $|\psi_{\text{target}}\rangle$.
Building on an idea of Yang and Navascués [1], our self-testing correlations will be block diagonal with 2×2 blocks. The tables for measurement settings $x, y \in \{0,1\}$ are given in Supplementary Tables 1 and 2 for even and odd d respectively. The 2×2 blocks $C_{x,y,m}$ are given by $(c_{2m}^2 + c_{2m+1}^2) \cdot C^{\text{ideal}}_{x,y,\theta_m}$ where $C^{\text{ideal}}_{x,y,\theta_m}$ is the 2-by-2 correlation table which maximally violates the tilted-CHSH inequality [2] which self-tests the state $\cos(\theta_m)|00\rangle + \sin(\theta_m)|11\rangle$, where $\theta_m := \arctan\left(\frac{c_{2m+1}}{c_{2m}}\right) \in (0, \frac{\pi}{2})$. They are given precisely in Supplementary Tables 3-5, with $\mu_m := \arctan(\sin(2\theta_m))$. The correlation tables for measurement settings $x \in \{0,2\}, y \in \{2,3\}$, are presented later in Supplementary Tables 6-10, after having derived some useful consequences of the correlations of measurement settings $x, y \in \{0,1\}$.

The proof of self-testing goes through constructing operators satisfying the sufficient conditions from the criterion of Yang and Navascués [1], presented in the main text. We will then argue that the same local isometry Φ that is guaranteed to exist by the criterion suffices to self-test the ideal measurements (described precisely in subsection D).

Recall that $\Pi_i^{A_x}$ is the projection corresponding to Alice obtaining outcome i on measurement setting x, and similarly for $\Pi_i^{B_y}$ on Bob's side. We need not assume that Alice and Bob's joint state is pure, but rather we take it to be such for ease of exposition. It is easy to see that the proof goes through in precisely the same way for a general mixed state ρ, replacing the usual inner product with the Hilbert-Schmidt inner product. We define the measurement operators $\hat{A}_{x,m} = \Pi_{2m}^{A_x} - \Pi_{2m+1}^{A_x}$ and $\hat{B}_{y,m} = \Pi_{2m}^{B_y} - \Pi_{2m+1}^{B_y}$. Clearly, $(\hat{A}_{x,m})^2 = \Pi_{2m}^{A_x} + \Pi_{2m+1}^{A_x} := \mathbb{1}_m^{A_x}$ and

Supplementary Table 1: $T_{x,y}$ for $x, y \in \{0,1\}$ for even values of $d \geq 2$

<table>
<tr><th>$a\backslash b$</th><th>0</th><th>1</th><th>2</th><th>3</th><th>$\cdots$</th><th>$d-2$</th><th>$d-1$</th></tr>
<tr><td>0</td><td colspan="2" rowspan="2">$C_{x,y,m=0}$</td><td>0</td><td>0</td><td>$\cdots$</td><td>0</td><td>0</td></tr>
<tr><td>1</td><td>0</td><td>0</td><td>$\cdots$</td><td>0</td><td>0</td></tr>
<tr><td>2</td><td>0</td><td>0</td><td colspan="2" rowspan="2">$C_{x,y,m=1}$</td><td>$\cdots$</td><td>0</td><td>0</td></tr>
<tr><td>3</td><td>0</td><td>0</td><td>$\cdots$</td><td>0</td><td>0</td></tr>
<tr><td>$\vdots$</td><td>$\vdots$</td><td>$\vdots$</td><td>$\vdots$</td><td>$\vdots$</td><td>$\ddots$</td><td>$\vdots$</td><td>$\vdots$</td></tr>
<tr><td>$d-2$</td><td>0</td><td>0</td><td>0</td><td>0</td><td>$\cdots$</td><td colspan="2" rowspan="2">$C_{x,y,m=\frac{d}{2}-1}$</td></tr>
<tr><td>$d-1$</td><td>0</td><td>0</td><td>0</td><td>0</td><td>$\cdots$</td></tr>
</table>

Supplementary Table 2: $T_{x,y}$ for $x, y \in \{0, 1\}$ for odd values of $d \geq 3$

$a\backslash b$	0	1	2	3	$\cdots$	$d-3$	$d-2$	$d-1$
0	$C_{x,y,m=0}$		0	0	$\cdots$	0	0	0
1			0	0	$\cdots$	0	0	0
2	0	0	$C_{x,y,m=1}$		$\cdots$	0	0	0
3	0	0			$\cdots$	0	0	0
$\vdots$	$\vdots$	$\vdots$	$\vdots$	$\vdots$	$\ddots$	$\vdots$	$\vdots$	0
$d-3$	0	0	0	0	$\cdots$	$C_{x,y,m=\frac{d-3}{2}}$		0
$d-2$	0	0	0	0	$\cdots$			0
$d-1$	0	0	0	0	$\cdots$	0	0	c_{d-1}^2

Supplementary Table 3: 2×2 block correlation table $C_{x=0,y=0,m}$ and $C_{x=0,y=1,m}$

$a\backslash b$	$2m$	$2m+1$
$2m$	$c_{2m}^2 \cos^2\left(\frac{\mu_m}{2}\right)$	$c_{2m}^2 \sin^2\left(\frac{\mu_m}{2}\right)$
$2m+1$	$c_{2m+1}^2 \sin^2\left(\frac{\mu_m}{2}\right)$	$c_{2m+1}^2 \cos^2\left(\frac{\mu_m}{2}\right)$

$(\hat{B}_{y,m})^2 = \Pi_{2m}^{B_y} + \Pi_{2m+1}^{B_y} := \mathbb{1}_m^{B_y}$.

Now, $\|\Pi_{2m}^{A_0}|\psi\rangle\| = \sqrt{\langle\psi|\Pi_{2m}^{A_0}|\psi\rangle} = \sqrt{\langle\psi|\Pi_{2m}^{A_0} \cdot \sum_{i=0}^{d-1} \Pi_i^{B_0}|\psi\rangle} = \sqrt{c_{2m}^2 \cos^2\left(\frac{\mu_m}{2}\right) + c_{2m}^2 \sin^2\left(\frac{\mu_m}{2}\right)} = c_{2m}$, and $\|\Pi_{2m+1}^{A_0}|\psi\rangle\| = c_{2m+1}$. With similar other calculations we deduce that

$$\|\mathbb{1}_m^{A_i}|\psi\rangle\| = \|\mathbb{1}_m^{B_j}|\psi\rangle\| = \sqrt{c_{2m}^2 + c_{2m+1}^2} \quad \forall i, j \in \{0, 1\}. \tag{1}$$

Moreover, notice that $\langle\psi|\mathbb{1}_m^{A_i}\mathbb{1}_m^{B_j}|\psi\rangle = c_{2m}^2 + c_{2m+1}^2 = \|\mathbb{1}_m^{A_i}|\psi\rangle\| \cdot \|\mathbb{1}_m^{B_j}|\psi\rangle\|$. Hence, by Cauchy-Schwarz, it must be the case that

$$\mathbb{1}_m^{A_i}|\psi\rangle = \mathbb{1}_m^{B_j}|\psi\rangle \quad \forall i, j \in \{0, 1\}. \tag{2}$$

By design, the correlations are such that

$$\langle\psi|\alpha_m \hat{A}_{0,m} + \hat{A}_{0,m}\hat{B}_{0,m} + \hat{A}_{0,m}\hat{B}_{1,m} + \hat{A}_{1,m}\hat{B}_{0,m} - \hat{A}_{1,m}\hat{B}_{1,m}|\psi\rangle = \sqrt{8 + 2\alpha_m^2} \cdot (c_{2m}^2 + c_{2m+1}^2) \tag{3}$$

where $\alpha_m = \frac{2}{\sqrt{1+2\tan^2(2\theta_m)}}$. As such, this is not a maximal violation of the tilted CHSH inequality (since $|\psi\rangle$ has unit norm). However, we can get around this by defining the normalised state $|\psi'_m\rangle = \frac{\mathbb{1}_m^{A_0}|\psi\rangle}{\sqrt{c_{2m}^2 + c_{2m+1}^2}}$. Since $\hat{A}_{i,m}|\psi\rangle = \hat{A}_{i,m}\mathbb{1}_m^{A_i}|\psi\rangle = \hat{A}_{i,m}\mathbb{1}_m^{A_0}|\psi\rangle$, and $\hat{B}_{i,m}|\psi\rangle = \hat{B}_{i,m}\mathbb{1}_m^{B_i}|\psi\rangle = \hat{B}_{i,m}\mathbb{1}_m^{A_0}|\psi\rangle$, by (2), then (3) implies

$$\langle\psi'_m|\alpha_m \hat{A}_{0,m} + \hat{A}_{0,m}\hat{B}_{0,m} + \hat{A}_{0,m}\hat{B}_{1,m} + \hat{A}_{1,m}\hat{B}_{0,m} - \hat{A}_{1,m}\hat{B}_{1,m}|\psi'_m\rangle = \sqrt{8 + 2\alpha_m^2} \tag{4}$$

Supplementary Table 4: 2×2 block correlation table $C_{x=1,y=0,m}$

$a\backslash b$	$2m$	$2m+1$
$2m$	$\frac{1}{2}(c_{2m}\cos\left(\frac{\mu_m}{2}\right) + c_{2m+1}\sin\left(\frac{\mu_m}{2}\right))^2$	$\frac{1}{2}(c_{2m+1}\cos\left(\frac{\mu_m}{2}\right) - c_{2m}\sin\left(\frac{\mu_m}{2}\right))^2$
$2m+1$	$\frac{1}{2}(c_{2m}\cos\left(\frac{\mu_m}{2}\right) - c_{2m+1}\sin\left(\frac{\mu_m}{2}\right))^2$	$\frac{1}{2}(c_{2m+1}\cos\left(\frac{\mu_m}{2}\right) + c_{2m}\sin\left(\frac{\mu_m}{2}\right))^2$

Supplementary Table 5: 2×2 block correlation table $C_{x=1,y=1,m}$

$a\backslash b$	$2m$	$2m{+}1$
$2m$	$\frac{1}{2}(c_{2m}\cos(\frac{\mu_m}{2}) - c_{2m+1}\sin(\frac{\mu_m}{2}))^2$	$\frac{1}{2}(c_{2m+1}\cos(\frac{\mu_m}{2}) + c_{2m}\sin(\frac{\mu_m}{2}))^2$
$2m{+}1$	$\frac{1}{2}(c_{2m}\cos(\frac{\mu_m}{2}) + c_{2m+1}\sin(\frac{\mu_m}{2}))^2$	$\frac{1}{2}(c_{2m+1}\cos(\frac{\mu_m}{2}) - c_{2m}\sin(\frac{\mu_m}{2}))^2$

Bamps and Pironio [3] proved that such a maximal violation of the tilted-CHSH inequality implies that, letting $\tilde{Z}_{A,m} := \hat{A}_{0,m}$, $\tilde{X}_{A,m} := \hat{A}_{1,m}$, $\tilde{\tilde{Z}}_{B,m} := \frac{\hat{B}_{0,m}+\hat{B}_{1,m}}{2\cos(\mu_m)}$, $\tilde{\tilde{X}}_{B,m} := \frac{\hat{B}_{0,m}-\hat{B}_{1,m}}{2\sin(\mu_m)}$, and then letting $\tilde{Z}_{B,m} := \frac{\tilde{\tilde{Z}}_{B,m}}{|\tilde{\tilde{Z}}_{B,m}|}$ and $\tilde{X}_{B,m} := \frac{\tilde{\tilde{X}}_{B,m}}{|\tilde{\tilde{X}}_{B,m}|}$, we have

$$\tilde{Z}_{A,m}|\psi'_m\rangle = \tilde{Z}_{B,m}|\psi'_m\rangle \tag{5}$$

$$\tilde{X}_{A,m}(\mathbb{1}_m^{A_0} - \tilde{Z}_{A,m})|\psi'_m\rangle = \tan(\theta_m)\tilde{X}_{B,m}(\mathbb{1}_m^{A_0} + \tilde{Z}_{A,m})|\psi'_m\rangle \tag{6}$$

Here, we are slightly abusing notation in $\frac{\tilde{\tilde{Z}}_{B,m}}{|\tilde{\tilde{Z}}_{B,m}|}$ and $\frac{\tilde{\tilde{X}}_{B,m}}{|\tilde{\tilde{X}}_{B,m}|}$, and hence we clarify how these quantities are defined. They are obtained via the following steps. First notice that all non-zero eigenvalues of $\tilde{\tilde{Z}}_{B,m}$ and $\tilde{\tilde{X}}_{B,m}$ necessarily correspond to eigenvectors in the subspace $\mathcal{B}_m = \text{range}(\mathbb{1}_m^{B_0}) + \text{range}(\mathbb{1}_m^{B_1})$. We divide these eigenvalues by their moduli. Then, we replace 0 eigenvalues with 1 if they correspond to eigenvectors in the subspace $\mathcal{B}_m$. The remaining 0 eigenvalues are left as they are. We remark that we defined the operators $\tilde{Z}_{B,m}$ and $\tilde{X}_{B,m}$ slightly differently than in [3], since we replaced, with 1, only the 0 eigenvalues corresponding to eigenvectors in $\mathcal{B}_m$, rather than *all* 0 eigenvalues, but it is clear that this change doesn't affect the conclusion of Bamps and Pironio [3] that we appealed to, since $|\psi'_m\rangle$ has no support outside of $\mathcal{B}_m$. As a consequence, $\tilde{Z}_{B,m}$ and $\tilde{X}_{B,m}$ are not unitary, and we have instead $(\tilde{Z}_{A,m})^2 = \mathbb{1}_m^{A_0}$, $(\tilde{X}_{A,m})^2 = \mathbb{1}_m^{A_1}$ and $(\tilde{Z}_{B,m})^2 = (\tilde{X}_{B,m})^2 = \mathbb{1}_{\mathcal{B}_m}$, where the latter is the projection on the subspace $\mathcal{B}_m$.

Note that, importantly, (5) and (6) also imply

$$\tilde{Z}_{A,m}|\psi\rangle = \tilde{Z}_{B,m}|\psi\rangle \tag{7}$$

$$\tilde{X}_{A,m}(\mathbb{1}_m^{A_0} - \tilde{Z}_{A,m})|\psi\rangle = \tan(\theta_m)\tilde{X}_{B,m}(\mathbb{1}_m^{A_0} + \tilde{Z}_{A,m})|\psi\rangle \tag{8}$$

and this is because $\tilde{Z}_{A,m}|\psi'_m\rangle = \frac{1}{\sqrt{c_{2m}^2+c_{2m+1}^2}}\tilde{Z}_{A,m}\mathbb{1}_m^{A_0}|\psi\rangle = \frac{1}{\sqrt{c_{2m}^2+c_{2m+1}^2}}\tilde{Z}_{A,m}|\psi\rangle$, and also

$$\tilde{Z}_{B,m}|\psi'_m\rangle = \frac{1}{\sqrt{c_{2m}^2 + c_{2m+1}^2}}\tilde{Z}_{B,m}\mathbb{1}_m^{A_0}|\psi\rangle = \frac{1}{\sqrt{c_{2m}^2 + c_{2m+1}^2}}\tilde{Z}_{B,m}|\psi\rangle \tag{9}$$

where we have used (2) and the fact that

$$\mathbb{1}_m^{B_0}|\psi\rangle = \mathbb{1}_m^{B_1}|\psi\rangle \implies \mathbb{1}_{\mathcal{B}_m}|\psi\rangle = \mathbb{1}_m^{B_i}|\psi\rangle\,. \tag{10}$$

Now, we similarly make the correlations $T_{x,y}$ between measurement settings $x \in \{0,2\}$ and $y \in \{2,3\}$ be also block-diagonal, but "shifted down" appropriately by one measurement outcome. The 2×2 blocks are $D_{x,y,m}$ (corresponding to outcomes $2m+1$ and $2m+2$) for $x \in \{0,2\}$ and $y \in \{2,3\}$, defined as $D_{x,y,m} := (c_{2m+1}^2 + c_{2m+2}^2) \cdot C^{ideal}_{x,y;\theta'_m}$, where $\theta'_m := \arctan\left(\frac{c_{2m+2}}{c_{2m+1}}\right) \in (0, \frac{\pi}{2})$. The correlations, $T_{x,y}$, for $x \in \{0,2\}$ and $y \in \{2,3\}$ are given by Supplementary Tables 6 to 10 where $\mu'_m := \arctan(\sin(2\theta'_m))$.

We can define the operators $\hat{A}'_{0,m} = \Pi^{A_0}_{2m+1} - \Pi^{A_0}_{2m+2}$, $\hat{A}'_{1,m} = \Pi^{A_2}_{2m+1} - \Pi^{A_2}_{2m+2}$ $\hat{B}'_{0,m} = \Pi^{B_2}_{2m+1} - \Pi^{B_2}_{2m+2}$, $\hat{B}'_{1,m} = \Pi^{B_3}_{2m+1} - \Pi^{B_3}_{2m+2}$, and $\mathbb{1}_m^{A'_x} = \left(\hat{A}'_{x,m}\right)^2$ and $\mathbb{1}_m^{B'_y} = \left(\hat{B}'_{y,m}\right)^2$. We also define the subspace $\mathcal{B}'_m = \text{range}(\mathbb{1}_m^{B'_0}) + \text{range}(\mathbb{1}_m^{B'_1})$. Using the argument employed earlier and following the same procedure, we can similarly construct operators $\tilde{Z}'_{A,m}$, $\tilde{X}'_{A,m}$, $\tilde{Z}'_{B,m}$ and $\tilde{X}'_{B,m}$ from operators $\hat{A}'_{x,m}$ and $\hat{B}'_{y,m}$ such that

Supplementary Table 6: $T_{x,y}$ for $x \in \{0,2\}$ and $y \in \{2,3\}$, for even values of $d \geq 2$

$a\backslash b$	1	2	3	4	$\cdots$	$d-1$	0
1	$D_{x,y,m=0}$		0	0	$\cdots$	0	0
2			0	0	$\cdots$	0	0
3	0	0	$D_{x,y,m=1}$		$\cdots$	0	0
4	0	0			$\cdots$	0	0
$\vdots$	$\vdots$	$\vdots$	$\vdots$	$\vdots$	$\ddots$	$\vdots$	$\vdots$
$d-1$	0	0	0	0	$\cdots$	$D_{x,y,m=\frac{d}{2}-1}$	
0	0	0	0	0	$\cdots$		

Supplementary Table 7: $T_{x,y}$ for $x \in \{0,2\}$ and $y \in \{2,3\}$, for odd values of $d \geq 3$

$a\backslash b$	1	2	3	4	$\cdots$	$d-2$	$d-1$	0
1	$D_{x,y,m=0}$		0	0	$\cdots$	0	0	0
2			0	0	$\cdots$	0	0	0
3	0	0	$D_{x,y,m=1}$		$\cdots$	0	0	0
4	0	0			$\cdots$	0	0	0
$\vdots$	$\vdots$	$\vdots$	$\vdots$	$\vdots$	$\ddots$	$\vdots$	$\vdots$	0
$d-2$	0	0	0	0	$\cdots$	$D_{x,y,m=\frac{d-3}{2}}$		0
$d-1$	0	0	0	0	$\cdots$			0
0	0	0	0	0	$\cdots$	0	0	c_0^2

Supplementary Table 8: 2×2 block correlation table $D_{x=0,y=2,m}$ and $D_{x=0,y=3,m}$

$a\backslash b$	$2m{+}1$	$2m{+}2$
$2m{+}1$	$c_{2m+1}^2 \cos^2(\frac{\mu'_m}{2})$	$c_{2m+1}^2 \sin^2(\frac{\mu'_m}{2})$
$2m{+}2$	$c_{2m+2}^2 \sin^2(\frac{\mu'_m}{2})$	$c_{2m+2}^2 \cos^2(\frac{\mu'_m}{2})$

Supplementary Table 9: 2×2 block correlation table $D_{x=2,y=2,m}$

$a\backslash b$	$2m{+}1$	$2m{+}2$
$2m{+}1$	$\frac{1}{2}(c_{2m+1}\cos(\frac{\mu'_m}{2}) + c_{2m+2}\sin(\frac{\mu'_m}{2}))^2$	$\frac{1}{2}(c_{2m+2}\cos(\frac{\mu'_m}{2}) - c_{2m+1}\sin(\frac{\mu'_m}{2}))^2$
$2m{+}2$	$\frac{1}{2}(c_{2m+1}\cos(\frac{\mu'_m}{2}) - c_{2m+2}\sin(\frac{\mu'_m}{2}))^2$	$\frac{1}{2}(c_{2m+2}\cos(\frac{\mu'_m}{2}) + c_{2m+1}\sin(\frac{\mu'_m}{2}))^2$

Supplementary Table 10: 2×2 block correlation table $D_{x=2,y=3,m}$

$a\backslash b$	$2m{+}1$	$2m{+}2$
$2m{+}1$	$\frac{1}{2}(c_{2m+1}\cos(\frac{\mu'_m}{2}) - c_{2m+2}\sin(\frac{\mu'_m}{2}))^2$	$\frac{1}{2}(c_{2m+2}\cos(\frac{\mu'_m}{2}) + c_{2m+1}\sin(\frac{\mu'_m}{2}))^2$
$2m{+}2$	$\frac{1}{2}(c_{2m+1}\cos(\frac{\mu'_m}{2}) + c_{2m+2}\sin(\frac{\mu'_m}{2}))^2$	$\frac{1}{2}(c_{2m+2}\cos(\frac{\mu'_m}{2}) - c_{2m+1}\sin(\frac{\mu'_m}{2}))^2$

$$\tilde{Z}'_{A,m}|\psi\rangle = \tilde{Z}'_{B,m}|\psi\rangle \tag{11}$$

$$\tilde{X}'_{A,m}(\mathbb{1}_m^{A'_0} - \tilde{Z}'_{A,m})|\psi\rangle = \tan(\theta'_m)\tilde{X}'_{B,m}(\mathbb{1}_m^{A'_0} + \tilde{Z}'_{A,m})|\psi\rangle \tag{12}$$

We remark that the correlations we described in Supplementary Tables 1-5 and 6-10 are indeed quantum correlations, meaning that they can be achieved by some measurements on a quantum state. In fact, they are naturally achieved when the joint state of the two provers is $|\psi_{\text{target}}\rangle$, and the observables on Alice and Bob's side are direct sums of 2×2 observables that are ideal for the appropriate tilted CHSH correlations (see subsection D).

B. Self-testing the overall state

Recall that we wish to prove the existence of a local isometry Φ such that $\Phi(|\psi\rangle) = |\text{extra}\rangle \otimes |\psi_{\text{target}}\rangle$, where the target state is given by $|\psi_{\text{target}}\rangle = \sum_{i=0}^{d-1} c_i|ii\rangle$ where $0 < c_i < 1$ for all i and $\sum_{i=0}^{d-1} c_i^2 = 1$.

The sufficient criterion for self-testing the state $|\psi_{\text{target}}\rangle$ from Yang and Navascués [1] is: the existence of a complete set of projectors $\{P_{A/B}^{(k)}\}$ and of unitary operators $X_{A/B}^{(k)}$ and $Z_{A/B}$ that satisfy the following for all $k = 0, 1, ..., d$:

$$P_A^{(k)}|\psi\rangle = P_B^{(k)}|\psi\rangle, \tag{13}$$

$$Z_{A/B} = \sum_{k=0}^{d-1} \omega^k P_{A/B}^{(k)}, \tag{14}$$

$$X_A^{(k)} P_B^{(k)}|\psi\rangle = \frac{c_k}{c_0}(X_B^{(k)})^\dagger P_A^{(0)}|\psi\rangle \tag{15}$$

where $\omega = e^{2\pi i/d}$. For completeness, the proof of this criterion is included in subsection E. In what follows, we will show how to construct such operators.

Inspired by [1], we start by defining the "flip" operators $X'_{A,m}$, $X'_{B,m}$, $Y'_{A,m}$ and $Y'_{B,m}$. Intuitively, the flip operator $X'_{A,m}$ will be a unitary operator whose role is to act on $P_A^{(2m+1)}|\psi\rangle$ (which is equal to $P_B^{(2m+1)}|\psi\rangle$ when condition (13) is satisfied) and turn it into $X'_{B,m}P_A^{(2m)}|\psi\rangle$, up to an appropriate factor. On the other hand, the flip operator $Y'_{A,m}$ will turn $P_A^{(2m)}|\psi\rangle$ into $Y'_{B,m}P_A^{(2m-1)}|\psi\rangle$, up to a factor. The idea is, then, that the appropriate alternating product of the unitary flip operators $X'_{A,m}$, $Y'_{A,m}$ will turn $P_A^{(i)}|\psi\rangle$ into precisely $\frac{c_i}{c_0}(X_B^{(i)})^\dagger P_A^{(0)}|\psi\rangle$, and we will let these alternating products be the $X_A^{(i)}$ and $X_B^{(i)}$ required by condition (15).

We define the X' flip operators as

$$X'_{A,m} = \tilde{X}_{A,m} + \mathbb{1} - \mathbb{1}_m^{A_1} \tag{16}$$

$$X'_{B,m} = \tilde{X}_{B,m} + \mathbb{1} - \mathbb{1}_{\mathcal{B}_m} \tag{17}$$

(Here we depart from the proof of [1]. In their definition of the flip operators, they use an operator $\mathbb{1}_m$ which is not uniquely defined when it does not act directly on the state). Clearly $X'_{A,m}$ and $X'_{B,m}$ are hermitian. They are also unitary by construction:

$$\begin{aligned}(X'_{A,m})^2 &= (\tilde{X}_{A,m})^2 + \tilde{X}_{A,m}(\mathbb{1} - \mathbb{1}_m^{A_1}) + (\mathbb{1} - \mathbb{1}_m^{A_1})\tilde{X}_{A,m} + \mathbb{1} - \mathbb{1}_m^{A_1} \\ &= (\tilde{X}_{A,m})^2 + \mathbb{1} - \mathbb{1}_m^{A_1} = \mathbb{1}_m^{A_1} + \mathbb{1} - \mathbb{1}_m^{A_1} = \mathbb{1}\end{aligned} \tag{18}$$

$$\begin{aligned}(X'_{B,m})^2 &= (\tilde{X}_{B,m})^2 + \tilde{X}_{B,m}(\mathbb{1} - \mathbb{1}_{\mathcal{B}_m}) + (\mathbb{1} - \mathbb{1}_{\mathcal{B}_m})\tilde{X}_{B,m} + \mathbb{1} - \mathbb{1}_{\mathcal{B}_m} \\ &= (\tilde{X}_{B,m})^2 + \mathbb{1} - \mathbb{1}_{\mathcal{B}_m} = \mathbb{1}_{\mathcal{B}_m} + \mathbb{1} - \mathbb{1}_{\mathcal{B}_m} = \mathbb{1}\,.\end{aligned} \tag{19}$$

Moreover, we still have

$$X'_{A,m}(\mathbb{1}_m^{A_0} - \tilde{Z}_{A,m})|\psi\rangle = \tan(\theta_m)X'_{B,m}(\mathbb{1}_m^{A_0} + \tilde{Z}_{A,m})|\psi\rangle\,. \tag{20}$$

Indeed, this follows from combining (6) with

$$\begin{aligned}(\mathbb{1}-\mathbb{1}_m^{A_1})(\mathbb{1}_m^{A_0}-\tilde{Z}_{A,m})|\psi\rangle &= (\mathbb{1}-\mathbb{1}_m^{A_1})(\mathbb{1}_m^{B_0}-\tilde{Z}_{B,m})|\psi\rangle \\ &= (\mathbb{1}_m^{B_0}-\tilde{Z}_{B,m})(\mathbb{1}-\mathbb{1}_m^{A_0})|\psi\rangle \\ &= (\mathbb{1}-\mathbb{1}_m^{A_0})(\mathbb{1}_m^{A_0}-\tilde{Z}_{A,m})|\psi\rangle = 0 \end{aligned} \tag{21}$$

and with

$$\begin{aligned}\big(\mathbb{1}-\mathbb{1}_{\mathcal{B}_m}\big)(\mathbb{1}_m^{A_0}+\tilde{Z}_{A,m})|\psi\rangle &= (\mathbb{1}_m^{A_0}+\tilde{Z}_{A,m})\big(\mathbb{1}-\mathbb{1}_{\mathcal{B}_m}\big)|\psi\rangle \\ &= (\mathbb{1}_m^{A_0}+\tilde{Z}_{A,m})(\mathbb{1}-\mathbb{1}_m^{B_0})|\psi\rangle \\ &= (\mathbb{1}_m^{A_0}+\tilde{Z}_{A,m})(\mathbb{1}-\mathbb{1}_m^{A_0})|\psi\rangle = 0 \end{aligned} \tag{22}$$

where the second last line uses (10). In particular, it follows from (20) that

$$X'_{A,m}\Pi_{2m+1}^{A_0}|\psi\rangle = \tan(\theta_m)X'_{B,m}\Pi_{2m}^{A_0}|\psi\rangle = \frac{c_{2m+1}}{c_{2m}}X'_{B,m}\Pi_{2m}^{A_0}|\psi\rangle \tag{23}$$

This concludes the description of the properties of the X' flip operators. Similarly, we define

$$Y'_{A,m} = \tilde{X}'_{A,m} + \mathbb{1} - \mathbb{1}_m^{A'_1} \tag{24}$$

$$Y'_{B,m} = \tilde{X}'_{B,m} + \mathbb{1} - \mathbb{1}_{\mathcal{B}'_m} \tag{25}$$

which are unitary, hermitian and satisfying

$$Y'_{A,m}\Pi_{2m+2}^{A_0}|\psi\rangle = \tan(\theta'_m)Y'_{B,m}\Pi_{2m+1}^{A_0}|\psi\rangle = \frac{c_{2m+2}}{c_{2m+1}}Y'_{B,m}\Pi_{2m+1}^{A_0}|\psi\rangle \tag{26}$$

C. Constructing the operators $X_{A/B}^{(k)}$ and $Z_{A/B}$

First, we need to define our $P_{A/B}^{(k)}$. Let $P_A^{(2m)} := (\mathbb{1}_m^{A_0}+\tilde{Z}_{A,m})/2 = \Pi_{2m}^{A_0}$, $P_A^{(2m+1)} := (\mathbb{1}_m^{A_0}-\tilde{Z}_{A,m})/2 = \Pi_{2m+1}^{A_0}$, $P_B^{(2m)} := (\mathbb{1}_{\mathcal{B}_m}+\tilde{Z}_{B,m})/2$ and $P_B^{(2m+1)} := (\mathbb{1}_{\mathcal{B}_m}-\tilde{Z}_{B,m})/2$. It holds, for $k=2m,2m+1$, that

$$\begin{aligned}P_A^{(k)}|\psi\rangle = (\mathbb{1}_m^{A_0}+(-1)^k\tilde{Z}_{A,m})/2|\psi\rangle &= (\mathbb{1}_m^{B_0}+(-1)^k\tilde{Z}_{B,m})/2|\psi\rangle \\ &= (\mathbb{1}_{\mathcal{B}_m}+(-1)^k\tilde{Z}_{B,m})/2|\psi\rangle = P_B^k|\psi\rangle \end{aligned} \tag{27}$$

where the last step uses (5). Hence, $P_A^{(k)}|\psi\rangle = P_B^{(k)}|\psi\rangle$ for $k=0,..,d-1$.
Then, let $Z_{A/B} := \sum_{i=0}^{d-1} w^i P_{A/B}^{(i)}$.
Next, we will define $X_{A/B}^{(k)}$ as follows:

$$X_A^{(k)} = \begin{cases}\mathbb{1}, & \text{if } k=0 \\ X'_{A,0}Y'_{A,0}X'_{A,1}Y'_{A,1}\ldots X'_{A,m-1}Y'_{A,m-1}X'_{A,m} & \text{if } k=2m+1 \\ X'_{A,0}Y'_{A,0}X'_{A,1}Y'_{A,1}\ldots X'_{A,m-1}Y'_{A,m-1}, & \text{if } k=2m\end{cases} \tag{28}$$

and

$$X_B^{(k)} = \begin{cases}\mathbb{1}, & \text{if } k=0 \\ X'_{B,0}Y'_{B,0}X'_{B,1}Y'_{B,1}\ldots X'_{B,m-1}Y'_{B,m-1}X'_{B,m} & \text{if } k=2m+1 \\ X'_{B,0}Y'_{B,0}X'_{B,1}Y'_{B,1}\ldots X'_{B,m-1}Y'_{B,m-1}, & \text{if } k=2m\end{cases} \tag{29}$$

Again, $X_A^{(k)}$ and $X_B^{(k)}$ are unitary since they are product of unitaries. Finally we need to check that (15) is met. For the case $k = 0$,

$$\begin{aligned} X_A^{(0)} P_B^{(0)} |\psi\rangle &= \mathbb{1} P_A^{(0)} |\psi\rangle \\ &= \frac{c_0}{c_0} X_B^{(0)} P_A^{(0)} |\psi\rangle. \end{aligned} \tag{30}$$

For $k = 2m + 1$,

$$\begin{aligned} X_A^{(k)} P_B^{(k)} |\psi\rangle &= X_A^{(k)} P_A^{(k)} |\psi\rangle \\ &= X'_{A,0} Y'_{A,0} X'_{A,1} Y'_{A,1} \dots X'_{A,m-1} Y'_{A,m-1} X'_{A,m} \Pi_{2m+1}^{A_0} |\psi\rangle \\ &\overset{(23)}{=} \frac{c_{2m+1}}{c_{2m}} X'_{A,0} Y'_{A,0} X'_{A,1} Y'_{A,1} \dots X'_{A,m-1} Y'_{A,m-1} X'_{B,m} \Pi_{2m}^{A_0} |\psi\rangle \\ &= \frac{c_{2m+1}}{c_{2m}} X'_{B,m} X'_{A,0} Y'_{A,0} X'_{A,1} Y'_{A,1} \dots X'_{A,m-1} Y'_{A,m-1} \Pi_{2m}^{A_0} |\psi\rangle \\ &\overset{(26)}{=} \frac{c_{2m+1}}{c_{2m}} \cdot \frac{c_{2m}}{c_{2m-1}} X'_{B,m} X'_{A,0} Y'_{A,0} X'_{A,1} Y'_{A,1} \dots X'_{A,m-1} Y'_{B,m-1} \Pi_{2m-1}^{A_0} |\psi\rangle \\ &= \frac{c_{2m+1}}{c_{2m}} \cdot \frac{c_{2m}}{c_{2m-1}} X'_{B,m} Y'_{B,m-1} X'_{A,0} Y'_{A,0} X'_{A,1} Y'_{A,1} \dots X'_{A,m-1} \Pi_{2m-1}^{A_0} |\psi\rangle \\ &= \dots \\ &= \frac{c_{2m+1}}{\cancel{c_{2m}}} \cdot \frac{\cancel{c_{2m}}}{\cancel{c_{2m-1}}} \dots \frac{\cancel{c_2}}{\cancel{c_1}} \cdot \frac{\cancel{c_1}}{c_0} X'_{B,m} Y'_{B,m-1} X'_{B,m-1} \dots Y'_{B,1} X'_{B,1} Y'_{B,0} X'_{B,0} \Pi_0^{A_0} |\psi\rangle \\ &= \frac{c_{2m+1}}{c_0} (X_B^{(k)})^\dagger P_A^{(0)} |\psi\rangle \end{aligned} \tag{31}$$

which is indeed (15) since $2m + 1 = k$. The case $k = 2m$ is treated similarly. This completes the construction of the local isometry Φ, from the criterion of Yang and Navascues. To conclude the proof of Theorem 1, we just need to show that the same isometry also self-tests the ideal measurements given precisely in subsection D. The proof is completed in subsection F.

Finally, we remark that for arbitrary non-maximally entangled two-qubit states, the set of correlations we presented are the only general ones known so far, but there may be others (for instance, for a specific non-maximally entangled state, another criterion based on Hardy's paradox is known [4]). However, for specific states, such as the maximally entangled pair of qubits, much more is known: in particular, all the possible correlations that use two dichotomic measurements per party [5]. Among these, all those where $A_0|\psi\rangle = B_0|\psi\rangle$ (in the notation of Ref [5], $\alpha_{00} = 0$) can be utilised in our scheme as the 2×2 correlation blocks to self-test the maximally entangled pair of qudits for any d, recovering the proof sketch suggested in [1]. As we mentioned earlier, in this case the structure of the $x = y = 0$ measurement is such that one can drop Bob's fourth measurement: maximally entangled states of arbitrary dimension can be self-tested within a $[\{3, d\}, \{3, d\}]$ Bell scenario.

D. Ideal measurements achieving the self-testing correlations

We provide here ideal measurements on $|\psi_{\text{target}}\rangle = \sum_{i=0}^{d-1} c_i |ii\rangle$ that achieve the self-testing correlations described above. For $x = 0$, Alice measures in the computational basis (i.e. in the basis $\{|0\rangle, |1\rangle, \cdots, |d-1\rangle\}$). For $x = 1$ and $x = 2$, she measures in the basis of eigenstates of the observable that is a direct sum of σ_x's defined between pairs of adjacent levels $(2m, 2m + 1)$ and $(2m + 1, 2m + 2)$ respectively, where σ_x is the usual Pauli-X matrix and $m \in \{0, 1, \cdots, \frac{d}{2} - 1\}$ for even values of d and $m \in \{0, 1, \cdots, \frac{d-3}{2}\}$ for odd values of d. (i.e. for $x = 1$, Alice measures in the basis $\left\{\frac{|0\rangle+|1\rangle}{\sqrt{2}}, \frac{|0\rangle-|1\rangle}{\sqrt{2}}, \frac{|2\rangle+|3\rangle}{\sqrt{2}}, \cdots, \frac{|d-2\rangle-|d-1\rangle}{\sqrt{2}}\right\}$ and for $x = 2$, Alice measures $\left\{\frac{|1\rangle+|2\rangle}{\sqrt{2}}, \frac{|1\rangle-|2\rangle}{\sqrt{2}}, \frac{|3\rangle+|4\rangle}{\sqrt{2}}, \cdots, \frac{|d-1\rangle-|0\rangle}{\sqrt{2}}\right\}$ for even values of d).

In a similar way, for $y = 0$ and $y = 1$, Bob measures in the basis of eigenstates of the observable that is a direct sum of single-qubit observables $\cos(\mu_m)\sigma_z + \sin(\mu_m)\sigma_x$ and $\cos(\mu_m)\sigma_z - \sin(\mu_m)\sigma_x$ respectively, defined between pairs of two adjacent levels $(2m, 2m + 1)$. Here $\mu_m = \arctan(\sin(2\theta_m))$, where $\theta_m = \arctan(\frac{c_{2m+1}}{c_{2m}})$, and σ_z is the Pauli-Z matrix. For $y = 2$ and $y = 3$, the same but over $(2m + 1, 2m + 2)$ pairings and with $\theta_m = \arctan(\frac{c_{2m+2}}{c_{2m+1}})$.

E. Proof of the Yang-Navascués self-testing criterion

We report here, for completeness, the proof that conditions (13)-(15) are sufficient to construct the desired local isometry, from the work of Yang and Navascués [1]. Notice, that the proof goes through in the same way for a general mixed joint state ρ in place of $|\psi\rangle$, when the criterion is naturally generalized to mixed states.

Proof: Define the local isometry

$$\Phi := (R_{AA'} \otimes R_{BB'})(\bar{F}_{A'} \otimes \bar{F}_{B'})(S_{AA'} \otimes S_{BB'})(F_{A'} \otimes F_{B'}) \tag{32}$$

where F is the quantum Fourier transform, $\bar{F}$ is the inverse quantum Fourier transform, $R_{AA'}$ is defined so that $|\phi\rangle_A|k\rangle_{A'} \mapsto X_A^{(k)}|\phi\rangle_A|k\rangle_{A'}\ \forall|\phi\rangle$, and similarly for $R_{BB'}$, and $S_{AA'}$ is defined so that $|\phi\rangle_A|k\rangle_{A'} \mapsto Z_A^k|\phi\rangle_A|k\rangle_{A'}\ \forall|\phi\rangle$, and similarly for $S_{BB'}$. We compute the action of Φ on $|\psi\rangle_{AB}|0\rangle_{A'}|0\rangle_{B'}$:

$$|\psi\rangle_{AB}|0\rangle_{A'}|0\rangle_{B'} \overset{F_{A'}\otimes F_{B'}}{\longrightarrow} \frac{1}{d}\sum_{k,k'}|\psi\rangle_{AB}|k\rangle_{A'}|k'\rangle_{B'} \tag{33}$$

$$\overset{S_{AA'}\otimes S_{BB'}}{\longrightarrow} \frac{1}{d}\sum_{k,k'}\left(\sum_j \omega^j P_A^{(j)}\right)^k \left(\sum_{j'} \omega^{j'} P_B^{(j')}\right)^{k'} |\psi\rangle_{AB}|k\rangle_{A'}|k'\rangle_{B'} \tag{34}$$

$$=\frac{1}{d}\sum_{k,k',j,j'} \omega^{jk}\omega^{j'k'} P_A^{(j)} P_B^{(j')}|\psi\rangle_{AB}|k\rangle_{A'}|k'\rangle_{B'} \tag{35}$$

$$=\frac{1}{d}\sum_{k,k',j,j'} \omega^{jk}\omega^{j'k'} P_A^{(j)} P_A^{(j')}|\psi\rangle_{AB}|k\rangle_{A'}|k'\rangle_{B'} \tag{36}$$

$$=\frac{1}{d}\sum_{k,k',j} \omega^{j(k+k')} P_A^{(j)}|\psi\rangle_{AB}|k\rangle_{A'}|k'\rangle_{B'} \tag{37}$$

$$\overset{\bar{F}_{A'}\otimes \bar{F}_{B'}}{\longrightarrow} \frac{1}{d^2}\sum_{k,k',j,l,l'} \omega^{j(k+k')}\omega^{-lk}\omega^{-l'k'} P_A^{(j)}|\psi\rangle_{AB}|l\rangle_{A'}|l'\rangle_{B'} \tag{38}$$

$$=\frac{1}{d^2}\sum_{k,k',j,l,l'} \omega^{k(j-l)}\omega^{k'(j-l')} P_A^{(j)}|\psi\rangle_{AB}|l\rangle_{A'}|l'\rangle_{B'} \tag{39}$$

$$=\sum_j P_B^{(j)}|\psi\rangle_{AB}|j\rangle_{A'}|j\rangle_{B'} \tag{40}$$

$$\overset{R_{AA'}\otimes R_{BB'}}{\longrightarrow} \sum_j X_B^{(j)} X_A^{(j)} P_B^{(j)}|\psi\rangle_{AB}|j\rangle_{A'}|j\rangle_{B'} \tag{41}$$

$$=\sum_j \frac{c_i}{c_0} X_B^{(j)} (X_B^{(j)})^\dagger P_A^{(0)}|\psi\rangle_{AB}|j\rangle_{A'}|j\rangle_{B'} \tag{42}$$

$$=\sum_j P_A^{(0)} \frac{c_j}{c_0} X_B^{(j)} (X_B^{(j)})^\dagger |\psi\rangle_{AB}|j\rangle_{A'}|j\rangle_{B'} \tag{43}$$

$$=\sum_j P_A^{(0)} \frac{c_j}{c_0} |\psi\rangle_{AB}|j\rangle_{A'}|j\rangle_{B'} \tag{44}$$

$$=\frac{1}{c_0} P_A^{(0)}|\psi\rangle_{AB} \otimes \sum_j c_j|j\rangle_{A'}|j\rangle_{B'} \tag{45}$$

$$=|\text{extra}\rangle \otimes |\psi_{\text{target}}\rangle \tag{46}$$

F. Self-testing the measurements

Not much work is required to extend self-testing to the measurement operators, using the same local isometry Φ, defined via the projections $P_{A/B}^{(k)}$ and the unitary operators $Z_{A/B}$ and $X_{A/B}^{(k)}$, as defined in the main text.

Consider $\hat{A}_{x,m} = \Pi_{2m}^{A_x} - \Pi_{2m+1}^{A_x}$ and $\hat{B}_{y,m} = \Pi_{2m}^{B_y} - \Pi_{2m+1}^{B_y}$. Let $A_{x,m}$,$B_{y,m}$ be the single-qubit ideal measurements achieving maximal violation of tilted CHSH on the (2m,2m+1) subspace, i.e. $A_{0,m} = \sigma_z$, $A_{1,m} = \sigma_x$, $B_{0,m} =$

$\cos(\mu_m)\sigma_z + \sin(\mu_m)\sigma_x$, $B_{1,m} = \cos(\mu_m)\sigma_z - \sin(\mu_m)\sigma_x$, with $\sigma_z = |2m\rangle\langle 2m| - |2m+1\rangle\langle 2m+1|$ and similar. We claim, first, that $\Phi(\hat{A}_{x,m}|\psi\rangle) = |\text{extra}\rangle \otimes A_{x,m}|\psi_{\text{target}}\rangle$ and $\Phi(\hat{B}_{y,m}|\psi\rangle) = |\text{extra}\rangle \otimes B_{y,m}|\psi_{\text{target}}\rangle$.
Following closely the proof in subsection E up to Equation (40), we have

$$\begin{aligned}
\Phi(\hat{A}_{x,m}|\psi\rangle) &= R_{AA'} \otimes R_{BB'} \sum_j P_B^{(j)} \hat{A}_{x,m}|\psi\rangle_{AB}|j\rangle_{A'}|j\rangle_{B'} \\
&= R_{AA'} \otimes R_{BB'} \Big(P_B^{(2m)} \hat{A}_{x,m}|\psi\rangle_{AB}|2m\rangle_{A'}|2m\rangle_{B'} + P_B^{(2m+1)} \hat{A}_{x,m}|\psi\rangle_{AB}|2m+1\rangle_{A'}|2m+1\rangle_{B'} \Big) \\
&= X_B^{(2m)} X_A^{(2m)} P_B^{(2m)} \hat{A}_{x,m}|\psi\rangle_{AB}|2m\rangle_{A'}|2m\rangle_{B'} + X_B^{(2m+1)} X_A^{(2m+1)} P_B^{(2m+1)} \hat{A}_{x,m}|\psi\rangle_{AB}|2m+1\rangle_{A'}|2m+1\rangle_{B'} \\
&= X_B^{(2m)} X_A^{(2m)} \Big(P_B^{(2m)} \hat{A}_{x,m}|\psi\rangle_{AB}|2m\rangle_{A'}|2m\rangle_{B'} + X'_{A,m} X'_{B,m} P_B^{(2m+1)} \hat{A}_{x,m}|\psi\rangle_{AB}|2m+1\rangle_{A'}|2m+1\rangle_{B'} \Big) \\
&= X_B^{(2m)} X_A^{(2m)} \frac{1}{c_{2m}} P_B^{(2m)} |\psi\rangle_{AB} \otimes A_{x,m} \big(c_{2m}|2m\rangle_{A'}|2m\rangle_{B'} + c_{2m+1}|2m+1\rangle_{A'}|2m+1\rangle_{B'} \big) \\
&= \frac{1}{c_0} P_A^{(0)} |\psi\rangle_{AB} \otimes A_{x,m}|\psi_{\text{target}}\rangle = |\text{extra}\rangle \otimes A_{x,m}|\psi_{\text{target}}\rangle
\end{aligned} \tag{47}$$

where the second-to-last line follows from the definitions of $X'_{A,m}$ and $X'_{B,m}$ in the main text, and from the proof, in [3], that maximal violation of the tilted CHSH inequality self-tests the ideal single-qubit measurements. One obtains analogous statements involving $\hat{A}'_{0/1,m} = \Pi^{A_{0/2}}_{2m+1} - \Pi^{A_{0/2}}_{2m+2}$ and $\hat{B}'_{0/1,m} = \Pi^{B_{2/3}}_{2m+1} - \Pi^{B_{2/3}}_{2m+2}$.
From the above, we deduce that the measurements of Alice and Bob on $|\psi\rangle$ are equivalent under Φ, to the ideal measurements described in subsection D on $|\psi_{\text{target}}\rangle$.

II. SUPPLEMENTARY REFERENCES

[1] Yang, T. H. & Navascués, M. Robust self-testing of unknown quantum systems into any entangled two-qubit states. *Phys. Rev. A* **87**, 050102 (2013).

[2] Acín, A., Massar, S. & Pironio, S. Randomness versus nonlocality and entanglement. *Phys. Rev. Lett.* **108**, 100402 (2012).

[3] Bamps, C. & Pironio, S. Sum-of-squares decompositions for a family of Clauser-Horne-Shimony-Holt-like inequalities and their application to self-testing. *Phys. Rev. A* **91**, 052111 (2015).

[4] Rabelo, R., Law, Y.Z. & Scarani, V. Device-independent bounds for Hardy's experiment *Phys. Rev. Lett.* **109** 180401 (2012).

[5] Wang, Y., Wu, X. & Scarani, V. All the self-testings of the singlet for two binary measurements. *New Journal of Physics* **18**, 025021 (2016).
